# Mobility Prediction Using a Weighted Markov Model Based on Mobile User Classification

**DOI:** 10.3390/s21051740

**Published:** 2021-03-03

**Authors:** Ming Yan, Shuijing Li, Chien Aun Chan, Yinghua Shen, Ying Yu

**Affiliations:** 1 State Key Laboratory of Media Convergence and Communication, Communication University of China, Beijing 100024, China; 2 School of Information and Communications Engineering, Communication University of China, Beijing 100024, China; li_sj@cuc.edu.cn (S.L.); shenwan@cuc.edu.cn (Y.S.); yuying@cuc.edu.cn (Y.Y.); 3 Insta-Wireless, Notting Hill, VIC 3168, Australia; chienac@unimelb.edu.au; 4 Department of Electrical and Electronic Engineering, The University of Melbourne, Parkville, VIC 3010, Australia

**Keywords:** mobility prediction, weighted Markov model, mobile user, user classification, mobile communication

## Abstract

The vast amounts of mobile communication data collected by mobile operators can provide important insights regarding epidemic transmission or traffic patterns. By analyzing historical data and extracting user location information, various methods can be used to predict the mobility of mobile users. However, existing prediction algorithms are mainly based on the historical data of all users at an aggregated level and ignore the heterogeneity of individual behavior patterns. To improve prediction accuracy, this paper proposes a weighted Markov prediction model based on mobile user classification. The trajectory information of a user is extracted first by analyzing real mobile communication data, where the complexity of a user’s trajectory is measured using the mobile trajectory entropy. Second, classification criteria are proposed based on different user behavior patterns, and all users are classified with machine learning algorithms. Finally, according to the characteristics of each user classification, the step threshold and the weighting coefficients of the weighted Markov prediction model are optimized, and mobility prediction is performed for each user classification. Our results show that the optimized weighting coefficients can improve the performance of the weighted Markov prediction model.

## 1. Introduction

Mobile cellular networks transport massive amounts of data. By systemically collecting and mining these data, valuable insights can be gained [1]. Such insights can help service providers to better design operating solutions and improve the mobile user experience [2]. By extracting user location information and service preference information contained in mobile communication data, a spatiotemporal mobile user behavior model can be established so that user behavior patterns can be predicted [3,4]. Effective mobility prediction enables service providers to predict user needs in advance, thereby optimizing network resources and reducing network congestion [5,6]. As a result, mobile users can obtain the information they need faster and enjoy a better service experience [7].

The results of user mobility prediction can be applied to various fields, such as early warning of congestion trends and urban traffic planning [8]. By analyzing and mining a large amount of user mobile data, user crowd portraits can be established according to user interests and preferences, which can be applied to personalized advertising push services to reduce the consumption of advertising services and prevent users from receiving excessive irrelevant information [9]. More importantly, user mobility prediction also contains research significance in the management and prevention of epidemic transmission [10] and smart tourism [11].

Commonly used mobility prediction models include association rule mining-based models [12,13,14], Markov chain-based models [15,16,17,18,19], and neural network-based models [20,21]. Association rule mining is based on the regularity and periodicity of the user itineraries, and location prediction is performed by mining the key stops and frequent routes of mobile users [12]. Markov chain-based models, on the other hand, use the previous state or additional past states to predict the next state. Each location of a user is treated as a state, and the Markov transition matrix is established by counting the transition probability between different locations in the time series. The effect of the historical trajectory on the next location can be mined through the transfer matrix. The Markov chain-based mobility prediction models have been widely used in location and mobility prediction [15,16]. In contrast, neural network-based models predict the user’s next location by mining the contextual correlation between user trajectory sequences [20]. A potential variable model based on historical mobility attention is proposed to predict the mobility of users. The variational encoding can capture the potential features in the user’s history trajectories and greatly improve the efficiency and prediction performance compared with recurrent networks [22]. Moreover, the mobile entropy has been used to measure the uncertainty of mobile users’ mobility pattern, and the mobile entropy can be used to assist the prediction model [23].

However, these methods are based on the data of all mobile users at an aggregated level. Due to the diversity of user behaviors, prediction algorithms based on all user data will affect the accuracy of predictions for specific groups of users and individual users.

To address the above challenges, this paper establishes user classification models based on different behavior patterns from real mobile communications data and uses machine learning techniques to classify users into four types. Then, by analyzing the characteristics of different user classifications, the optimal step threshold and weighting coefficients of the weighted Markov model for each user classification are determined. Finally, the weighted Markov model is used to predict the trajectories of different user classifications. Our contributions are threefold:

We analyze real mobile communications data and extract mobile user trajectories. The user’s mobility is represented by calculating their mobility trajectory entropy (MTE). We propose and develop a classification method based on user behavior patterns. Based on the features extracted from the user trajectories, users are classified into four classes by using machine learning methods.

We employ a weighted Markov model to separately predict the trajectory for four user classes. We propose a new method for determining weighting coefficients of the weighted Markov model by analyzing the characteristics of different types of users.

The rest of the paper is organized as follows: Section 2 discusses related work on different mobile user mobility prediction methods. In Section 3, we first analyze the key fields of real mobile communication data and extract the user trajectories. The mobile trajectory entropy is also introduced to conduct a preliminary analysis of the trajectory complexity of all users. In Section 4, we propose a user classification model and use machine learning algorithms to classify all users. Section 5 introduces the basic principles and corresponding algorithms of a Markov prediction method. By analyzing the characteristics of each type of user, the optimal step threshold and the weighting coefficients of weighted Markov models are proposed. Hence, user mobility is predicted by using the weighted Markov model. In addition, a detailed analysis of the prediction results is performed. Section 6 concludes the paper.

## 2. Related Work

The study of human mobility and movements has been investigated extensively. Based on mobile communication data, related studies have shown that the prediction accuracy of human behavior can reach 93%, which provides a theoretical basis for mobility prediction [24]. Common positioning methods include global positioning system (GPS) data-based positioning [25,26] and wireless fidelity (Wi-Fi) data-based positioning [27]. In addition to the data used in these positioning methods, mobile communication data are available at service providers’ data centers and cover a wide range, which are highly suitable for research on the mobility of a large range of people. With the rapid development of big data technologies and data mining in recent years, it has become possible to use mobile communication data to mine people’s movement patterns [28]. Many scholars have researched the characteristics of people’s mobile behavior from different perspectives. For example, mobile behavior prediction can be used to solve network traffic congestion problems. Predicting traffic flow through mobile big data analysis can be exploited in a wide range of potential applications to make a city smarter and safer and can help reduce congestion and pollution [29,30]. In addition, the prediction of user mobility and the content request model can help to transfer and cache mobile content that users need in advance using wireless edge caches close to the users, thereby improving the user experience [31]. From epidemic modelling to self-driving vehicles and urban planning, we also need to build prediction models of human mobility [32,33]. A prediction model based on aggregated mobile phone call data can be well used in urban planning and disaster management [34,35].

Among different user location prediction methods, one of the basic methods predicts a user’s future location based on association rule mining. A trajectory prediction method based on frequent location patterns has been proposed. By creating a location pattern tree to store the sequence of regions that users frequently visit, this method greatly reduces the search space [12]. In addition, location prediction based on neural networks is also a commonly used method. Neural networks based on the Bayesian principle can integrate complex output distributions to achieve complex trajectory predictions. Bayesian recurrent neural network (RNN) models are typically used to ensure the long-term stability of autonomous driving and the flexibility of physical location predictions [21]. Relevant studies have shown that applying the long-term short-term memory (LSTM) network to key components of specific deep learning network can improve the generalization ability of the network and effectively reduce the error accumulation effect for multi-step prediction [36].

In addition to the above two methods, the most commonly used models are Markov chain-based prediction models because the Markov model can better represent human movements in time series. It constructs a crowd density prediction model and then realizes the prediction of user mobility patterns [15,16]. Based on the general Markov model, many studies have effectively improved the Markov model, which has greatly improved its prediction performance. A mobility prediction method that combines the user’s long-term and short-term trajectories has been proposed. The method uses a long-term trajectory to train a Markov process and uses the short-term trajectory to predict the user’s current possible random behavior. The accuracy of the next location prediction based on this model has been proven to reach more than 70% [37]. Another location prediction method combining a variable-order Markov model and the spatiotemporal law of the user has been proposed [17]. The Markov order is determined based on the matching between the current trajectory and the historical trajectories. This model improves the accuracy of location prediction, and it is especially suitable for cases where the user’s historical trajectory data are small [17]. Due to the increase in time and space complexity of higher-order Markov models, other variable-order Markov models have been proposed [18]. In addition, to improve the prediction accuracy of non-Gaussian mobility data, a hybrid Markov-based model has been proposed [19], and a weighted Markov model has been trained for near-term driving direction prediction [38]. To solve the problem of data sparsity in Markov chain-based prediction model, a sparsity trajectory prediction algorithm based on multiple entropy measures was proposed and the algorithm obtained a gain in prediction accuracy [39].

Trajectory prediction models based on mobile communication data have received a lot of attention. For example, the mobile patterns of users can be inferred from aggregated mobile phone call data to predict human mobility patterns [34]. Some researchers have established a framework of personal mobile pattern mining based on mobile phone location information [35]. In addition, to solve the sparsity problem of mobile communication data, a second-order Markov model with time dimension is used to predict the sparse trajectory [39]. However, most of the current user behavior analysis and mobility prediction algorithms based on cellular networks have been proposed based on the general mobile characteristics of all users. These methods usually ignore individual behavior differences between users and result in ineffective prediction methods and a certain waste of resources. To further improve the accuracy of trajectory prediction, unlike these existing works, we first analyze real mobile user data and classify mobile users into different categories based on the behavior characteristics of these users. Based on user classification using machine learning algorithms, different prediction methods are adopted for different user classifications, thereby achieving higher prediction accuracy.

## 3. Trajectory Extraction and Complexity Analysis 

In daily life, most people carry mobile phones with them most of the time. Mobile communication data basically include the location information of all users, and the rapid increase in the number of mobile devices has also greatly improved the coverage ratio. When a mobile device is connected to the mobile network, it needs to access the corresponding cellular base station. Therefore, by collecting and analyzing mobile communication data, it is possible to locate the mobile user and extract the movement trajectory. The positioning accuracy based on mobile communication data depends on the size of the cell radius, continuity of user reports, etc., and the inter-dependency between them has been investigated in detail in the minimization of drive test (MDT)-based location estimation method [40,41].

### 3.1. Data Acquisition and Data Format

Deep packet inspection (DPI) is the main signaling acquisition interface of the long-term evolution (LTE) core network. By detecting traffic and message content and filtering the traffic according to relevant rules, DPI devices can implement functions such as business traffic analysis, business traffic percentage statistics, and service identification of the associated link [42]. The data collected by the DPI collection system is called external data recording (XDR), which is developed from traditional call detail record (CDR) data. The XDR data are a detailed record of the signaling and services generated by processing all data [42]. The CDR log records various call information of the user, including the mobile phone number, the person called, the start and end times, the cell identification (ID) that provides the call service, and whether the caller is roaming. The XDR usually refers to the information recorded in data traffic from the mobile network. Each time a user conducts a session, an XDR record is generated [2].

We use real communications XDR data from China Mobile, which includes all networking data of 5000 users in three consecutive weeks. These XDR data are collected from the DPI device of Gn interface of China Mobile core network and include multiple fields such as mobile user ID, recording time, location area code (LAC), cell ID code, service type, and uplink and downlink traffic [42]. To protect the privacy of our users, the data we get is encrypted in advance. For example, the user ID in XDR data is not a real phone number, but rather a series of virtual numbers for every user. The LAC is the internationally unique identifier for each public land mobile network used for location updating of mobile subscribers, and the cell ID code represents the identification code of the connected cell within the base station. Combining the two codes, the user’s current location can be determined. The fields related to time and location are mainly used in our mobility prediction model.

### 3.2. Trajectory Extraction

From the perspective of trajectory modeling, the trajectory of a mobile user is the user’s location information, which changes over time and should include specific spatial and temporal information. Spatial information is a geographic attribute, such as a mobile base station, that a user has passed through during their movements, and temporal information can be represented by the time when the user arrives in or leaves an area, represented by the connection and disconnection of a user’s mobile device to the base station. Both spatial and temporal information can be represented by numerical features.

The user trajectory based on mobile network data records the location information of the base station to which the user is connected. Therefore, the real-time location of the user can be determined by the base station number in the record, which is a stop point in the user trajectory. In the XDR data, the location information of the base station to which the mobile user is connected is represented by the location area code, *LAC*, and the cell identification number, *Cell_ID* [2]. Since the longest digit of *Cell_ID* is 4 digits, the following equation can combine these two fields to uniquely indicates the user’s stay point, *S*:

(1) S=LAC×10000+Cell_ID,

The XDR data records of each user are processed on a daily basis, and the time of a day is divided into 288 time periods with a 5-min time window, which is the default setting in the XDR data collecting system. The cell location with the longest stay time within every 5-min time window is selected as the user’s current stay point information. If two successive stop points are the same, they are combined into one stop point. In the daily record, the user will have multiple stop points, and the multiple stop points are connected to form a user’s movement trajectory consisting of a series of stop points, as indicated by:

(2) $$Traj_{d}=\{S_{1},S_{2},\cdot \cdot \cdot ,S_{i},\cdot \cdot \cdot ,S_{n}\},$$

where *Traj*_d_ is the user’s movement trajectory in the selected day, *d* (1≤d≤21), and $S_{i}$ is a stop point in the trajectory at time window $t_{i}$. For a selected user $U_{j}$, the complete set of trajectories in 21 days can be expressed as:

(3) $$Traj(U_{j})=\left\{\;Traj_{1},Traj_{2},\cdot \cdot \cdot ,Traj_{d},\cdot \cdot \cdot ,Traj_{21}\right\},$$

### 3.3. Trajectory Complexity Analysis

In information theory, statistical physics and other disciplines, entropy is often used to measure the inherent chaos of a system. When studying the spatial movement behavior of users, the concept of entropy is adopted to measure the complexity of user trajectories, which is called MTE [43]. A larger MTE indicates that the user’s next location is very uncertain, that is, the trajectory is more complicated. A smaller MTE indicates that the user’s mobile behavior is more regular and has a more fixed behavior pattern.

The specific calculation steps of MTE are as follows:

- Divide the day into 24 periods in hours, $t_{i},\;i=1,2,3,\cdot \cdot \cdot ,24$, and count all cell sites visited by users in each hour, $Cell_{ij},\;j=1,2,3,\cdot \cdot \cdot ,n$.
- Calculate the prior probability of users visiting different cell sites $p_{ij}$ during the period $t_{i}$ using the following equation:
  (4) $$p_{ij}=\frac{T_{ij}}{T_{i}},$$
  where $T_{i}$ is the total duration of the time period, and $T_{ij}$ indicates the length of time the user stays at the cell site $Cell_{ij}$ during the time period $t_{i}$.

(1) The user’s MTE during period $t_{i}$ can be calculated as:
  (5) $$H(t_{i})=\sum_{j=1}^{n}p_{ij}\log(p_{ij}),$$
(2) Add the MTEs of all time periods to obtain the total MTE of the user for one day:
  (6) $$H_{day}=\sum_{i=1}^{24}H(t_{i}),$$

**Figure 1:**
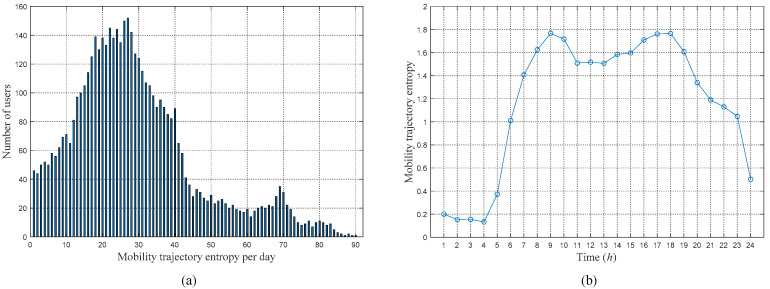
MTEs of all users: (**a**) Average daily MTE distribution of all users, (**b**) Average MTE in every hour of all users.

Using the above method, the MTEs of all users are calculated using MATLAB R2016a, and the statistical result is shown in Figure 1. Figure 1a shows the average daily MTE distribution of all users. It is observed that the daily MTEs of most users are relatively small and are mainly concentrated between 1 and 50. These users account for approximately 87.42% of the total number of users. This percentage shows that most users usually have relatively small ranges of activity and relatively simple movement trajectories. However, a small number of users have high mobile entropy, up to approximately 90. This value indicates that there are no regular patterns in the movements of these users and that their trajectories are relatively complicated. Figure 1b shows the average MTE of all users in different time periods within a day. It is observed that the distribution of user MTE per hour is similar to that of the user’s daily mobility. The peaks are concentrated in the morning and evening rush hours. This concentration shows that there is a positive correlation between the user’s movement intensity and the trajectory complexity.

## 4. Classification of Mobile Users Based on Behavior Patterns

The XDR data contain not only information on the cell sites that the user accesses but also the service information and traffic information the user accesses. The mobile users can thus be classified according to different criteria. For example, users can be divided into video preference users, social applications preference users, and web page preference users according to the analysis of service patterns. Users can also be divided into low traffic users and high traffic users based on traffic information. Here, to facilitate the prediction of user trajectories, we divide the users into four classifications according to user movement patterns and trajectory complexity.

### 4.1. User Classification Criteria

In daily life, some people spend most of their time at home, such as retired people and housewives. The data will show that they do not move much. Apart from these groups of mobile users, most other groups of mobile users will have to commute to work. Therefore, users can be simply divided into resident and nonresident categories. Among the nonresident category, due to different working patterns, their mobility characteristics also vary greatly. Some mobile users commute to work by public transportation every day, so their movement trajectory is more regular. However, some mobile users have more flexible work styles and therefore do not need to go to work every day or move around during work, such as postmen. Therefore, nonresident users can be divided into two types: regular commuters and irregular commuters. There are also some nonresidents whose work styles are different, such as night workers. These people are defined as other types of nonresidents. Table 1 shows the types of users and their characteristics. Since the data we obtained only covers the business district of a city in geographical scope, users are simply divided into the above four categories according to their mobile characteristics. It should be noted that there are different classification criteria in different application scenarios.

The user trajectory information extracted above is composed of the locations of the cell sites with time stamps. To analyze the movement patterns of different types of users from the trajectory information, it is necessary to extract the behavior characteristics hidden in the trajectory information as comprehensively and accurately as possible. The characteristics of different dimensions are defined to build a machine learning data set. Some contextual information that represents time and user location is used to determine the type of user. The parameters and corresponding descriptions extracted from the user data set are shown in Table 2.

The user type is mainly determined by the number of cell sites and the dwell time that the user trajectory contains over different time periods. The number of cell sites in different time periods reflects the activity pattern of a user to a certain extent. For example, if a user is a regular commuter, then he should work in a fixed location during working hours, so the number of cell sites (which reflects the movement) in working hours should be small. In addition, the length of dwell time also represents the mobility pattern of the user. If a user is continuously fixed at a cell site for more than 1 h, it is counted as a dwell, and the dwell time of each unique cell site is the sum of the lengths of each single dwell period at this cell site. For example, the length of resident time during the working time is the maximum of the dwell time of all unique cell sites in the corresponding period.

When extracting the contextual information, the trajectory information in the working day is selected and divided into different groups with 5 time periods in a day, such as morning peak (7:00–10:00), working hours (10:00–17:00), evening peak (17:00–21:00), evening activity time (21:00–23:00), and night-time (23:00–7:00). Then, the number of unique cell sites and the dwell time in each period are counted, and the average number within a week is used as the final value of the corresponding parameter. To label the selected users into categories so that they can be classified by machine learning methods, certain classification criteria need to be established. Based on the preliminary analysis and statistics of the real mobile data, we developed corresponding classification criteria, as shown in Table 3. There are no general principles for the classification criteria in the table. These values are set based on experience and can be adjusted according to the classification accuracy of machine learning methods.

### 4.2. Classification Method Evaluation

According to the classification criteria shown in Table 3, some users are classified by manual analysis. To evaluate the performance of user classification using different machine learning algorithms, a total of 200 user machine learning datasets are first constructed, with 60% of the labeled users selected as the training set and the remaining 40% used as the test set, and then the trained model is used to classify all other users. The naïve Bayes, decision tree and K-nearest neighbor (KNN) algorithms in the scikit-learn toolkit [44] are used as machine learning classification models, and the training set is used to train the classification models. The parameters use in our experiment are set to default values in the scikit-learn toolkit [44]. For examplene, in the naïve Bayes method, the prior probabilities of each category are not given, but statistics are carried out according to the actual situation of training data. The maximum number of features in the decision tree uses the number of all features. To compare the performance of different machine learning algorithms, the precision, recall, F1-score and overall accuracy of the different classification models are calculated. The experimental results are shown in Table 4.

The classification results in Table 4 show that the overall accuracy of the naive Bayes model is the lowest: 82.5%. The performances of the irregular commuters and others are relatively low, and the precision and recall are both below 80%. The overall classification accuracy based on the decision tree is higher than that of the naive Bayes model, reaching 92.5%. From the classification results of each type of user, its precision, recall and F1-score are relatively high. Because the KNN model is suitable for classification problems and is good at classifying rare events, the KNN model has better performance in user behavior feature classification compared to naive Bayes and decision tree models. It is also observed that the KNN model has the highest overall classification accuracy, as high as 95%. The classification precision, recall and F1-score of each user classification of the KNN model are above 90%. Therefore, the KNN classification model is selected to classify all users.

### 4.3. Classification Result

The results of KNN user classification are shown in Table 5. It is observed that regular commuters account for the highest proportion among all user classifications, i.e., approximately one-third of all users. The proportions of other types of users are above 20%. The classification results show that the distribution of each type of user is relatively uniform.

**Figure 2:**
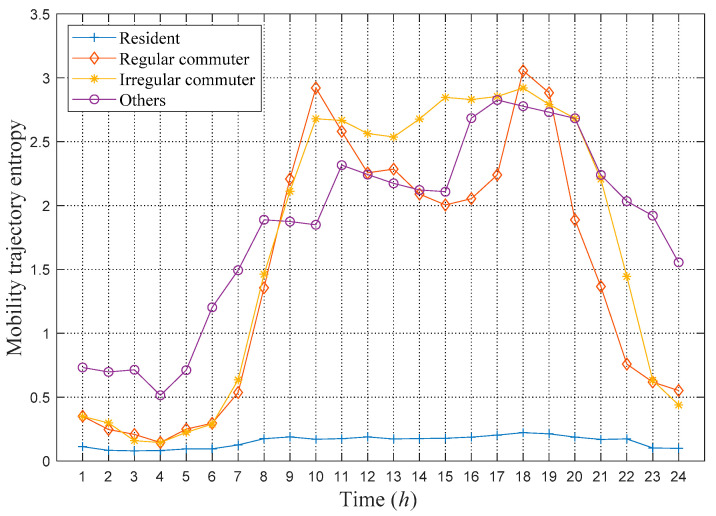
Average MTE in every hour of the four types of users.

To further verify the classification effect and understand the complexity of the movement trajectory of various types of users, the average MTEs in every hour of the four types of users are calculated using the method introduced in the previous chapter. The results are shown in Figure 2. It is observed that there are obvious differences in the mobility behavior of various types of users. Residents have lower MTE throughout the day, indicating that these users rarely move throughout the day. The MTE value of regular commuters presents a distinct double-peak characteristic, which indicates that these users commute to work on time every day, and they move very little during working hours. The MTE value of irregular commuters shows that their trajectories are more complicated in the daytime, and there will be more movements during working hours. Compared to the other three types of users, the category of others has higher entropy values at night, indicating that they move more frequently at night. The obvious differences in mobile features between each type of user also verify the rationality of the classification criteria and the effectiveness of the classification algorithm.

It should be noted that unsupervised learning algorithms have been applied to mobile user clustering in many existing studies [45,46,47], and their classification performance has been verified. Since the focus of this paper is the Markov prediction algorithm, we have not analyzed the effect of unsupervised learning algorithms at this stage.

## 5. Mobility Prediction Based on Weighted Markov Model

As described in the previous sections, the Markov model is widely used in location prediction. It uses the historical visit location sequence of the user to mine its transfer rule between each visit location, thereby predicting the user’s next location. The basic Markov model is a first-order Markov model, that is, the next location is determined only by the current location. This model does not actually conform to the basic laws of the moving process of moving objects. The high-order Markov model considers that the state of the next moment is related not only to the state of the current moment but also to the state of previous *k-1* moments. The next location of the mobile user is related to the locations at multiple previous moments. This assumption can improve the accuracy of the prediction model to a certain extent. However, the Markov model’s order cannot be as high as possible because the user’s mobile behavior does not have complete periodicity [48]. When the order exceeds a certain value, the computational complexity will increase significantly. 

### 5.1. Markov Chain

The Markov prediction model is based on the Markov chain, which is a kind of memory-free discrete-time random process [15,48]. Under the condition of a given moment, the state of the future at any moment is only related to the state of the current moment and has nothing to do with all states before the current moment. The definition of a Markov chain is as follows: for a discrete random process with Markov properties, let the state space $\left\{X_{n}:n=0,1,2,\cdot \cdot \cdot \right\}$ be finite; then, $X_{n}=i$ indicates that the object is in state *I* at time *n*. If for any positive integer *n*, the following equation is true:

(7) $$P(X_{n+1}=x{|X}_{1}=x_{1},\cdot \cdot \cdot ,X_{n}=x_{n})=P(X_{n+1}=x|X_{n}=x_{n}),$$

Then, such a stochastic process is called a Markov chain [15,16].

The core part of the Markov prediction model is the establishment of the transition probability matrix. Transition probability refers to the probability of the current state transitioning to the next state in the Markov chain. A matrix composed of transition probability is called a transition probability matrix. The number of transition steps is divided into one step or multiple steps. Similarly, the transition probability matrix is also divided into a one-step transition probability matrix and a *k*-step transition probability matrix. The one-step transition probability can be expressed as Equation (8), and the k-step transition probability can be expressed as Equation (9):

(8) $$P_{i,j}=P(X_{n+1}=x_{j}|X_{n}=x_{i}),$$

(9) $$P_{i,j}^{\left(k\right)}=P(X_{k}=x_{j}|X_{0}=x_{i}),$$

The one-step transition probability is arranged in a matrix form, and the one-step transition probability matrix is obtained. The one-step transition probability matrix is expressed as follows:

(10) $$P=\left[\begin{array}{ccccc} P_{1,1} & \cdot \cdot \cdot & P_{1,j} & \cdot \cdot \cdot & P_{1,m}\\ \vdots & \ddots & \vdots & ⋰ & \vdots \\ P_{i,1} & \cdot \cdot \cdot & P_{i,j} & \cdot \cdot \cdot & P_{i,m}\\ \vdots & ⋰ & \vdots & \ddots & \vdots \\ P_{m,1} & \cdot \cdot \cdot & P_{m,j} & \cdot \cdot \cdot & P_{m,m} \end{array}\right],$$

In the probability transition matrix *P*, the column element represents the possible next location of the moving object, and the row element represents the probability that it may be transferred to the location of the column element. When predicting the next location, we can query the corresponding column elements in the probability transition matrix and use the location with the highest probability as the prediction result.

Correspondingly, the *k*-step transition probability is arranged in a matrix form to obtain the *k*-step transition probability matrix, which is expressed as:

(11) $$P^{\left(k\right)}=\left[\begin{array}{ccccc} P_{1,1}^{\left(k\right)} & \cdot \cdot \cdot & P_{1,j}^{\left(k\right)} & \cdot \cdot \cdot & P_{1,m}^{\left(k\right)}\\ \vdots & \ddots & \vdots & ⋰ & \vdots \\ P_{i,1}^{\left(k\right)} & \cdot \cdot \cdot & P_{i,j}^{\left(k\right)} & \cdot \cdot \cdot & P_{i,m}^{\left(k\right)}\\ \vdots & ⋰ & \vdots & \ddots & \vdots \\ P_{m,1}^{\left(k\right)} & \cdot \cdot \cdot & P_{m,j}^{\left(k\right)} & \cdot \cdot \cdot & P_{m,m}^{\left(k\right)} \end{array}\right],$$

The *k*-step transition probability matrix can be obtained from the one-step transition probability matrix. The relationship between the two can be expressed as:

(12) $$P^{\left(k\right)}=P^{k},$$

Hence, we can find the *k*-step transition probability matrix through matrix multiplication. If k is large, it may be more convenient to compute *P(k)* via eigendecomposition [49].

### 5.2. Weighted Markov Prediction Model Based on Different User Types

The weighted Markov model is a hybrid Markov model. It uses weights to represent the impact of different order Markov models on the prediction results and then determines the final prediction result by weighted summation [38].

The key step in the weighted Markov model is the determination of the weighting coefficients. In general, for a user’s location prediction, the location closer to the current location has a greater degree of influence on the next location. Therefore, related studies have pointed out that low-order Markov models should have higher weights, and the weighting coefficients from step 1 to step *k* decrease in order [38]. A simple weighting coefficient calculation method is proposed as follows:

(13) $$\omega_{i}=\frac{k-i+1}{\sum_{m=1}^{k}m}\;,\;i=1,2,\cdot \cdot \cdot ,k,$$

where $\sum_{m=1}^{k}m$ is the sum of steps from 1 to *k*. Although this method is more reasonable, it does not take into account the actual mobility patterns of different scenes. In practical applications, appropriate weight determination methods should be formulated for different sample data so that the prediction accuracy will be higher.

By analyzing the characteristics of different types of users, this paper proposes a new method for determining weighting coefficients. In this method, the transition probability for different steps is directly proportional to the weighting coefficient, thereby highlighting the impact of the high transition probability on the prediction result. The specific calculation steps are as follows:

- Calculate the transition probability $p_{i}$ of each location in the historical trajectory to the next location: From the user’s historical trajectory, the one-step transition probability matrix *P* is established, and then the transition probability matrix $P^{\left(2\right)},\cdot \cdot \cdot ,P^{\left(k\right)}$ from 2 to *k* steps is obtained by Equation (12). We then use each transition probability matrix to predict the next location of the user and obtain the transition probability, $p_{i}(1\leq i\leq k)$, from the previous *k-1* location to the next location after *k* steps.
- Determine the maximum number of steps *k* of the weighted Markov model: For different types of users, different historical positions have different degrees of influence on the next position. This paper defines a probability threshold $P_{th}$ and a step threshold $L_{th}$ to jointly determine the value of *k*. The probability threshold $P_{th}$ is used to filter out the impact of higher-order Markov models with lower prediction probability on location prediction. As *k* increases, the *k*-step transition probability gradually decreases. When the transition probability is less than $P_{th}$, no more steps are considered. The step threshold $L_{th}$ is used to eliminate the effect of higher steps on the prediction. In the weighted Markov model, the number of steps greater than $L_{th}$ is no longer considered. The specific values of $P_{th}$ and $L_{th}$ are set according to the actual situation. The maximum number of steps *i* used in the actual weighted Markov model should be less than or equal to $L_{th}$, and the *i*-step transition probability $p_{i}$ is greater than or equal to $P_{th}$. The maximum number of steps *k* is the maximum value of all *i* that meet the requirements, which is expressed as follows:
  (14) $$k=\max(i),\;(i\leq L_{th})\&(p_{i}\geq P_{th}),$$
- Calculate the weighting coefficient $\omega_{i}$: After *k* is determined, the weighting coefficients of different orders of Markov models are determined by the transition probability of the corresponding number of steps. The higher the transition probability is, the larger the weighting coefficient. The weighting coefficient can be calculated as:
  (15) $$\omega_{i}=p_{i}/\sum_{m=1}^{k}p_{m}\;,\;i=1,2,\cdot \cdot \cdot ,k,$$
- Calculate the probability of moving to all possible next positions: Assuming the current location is *S*_1_ and the previous *k-1* locations are $S_{2},S_{3},\cdot \cdot \cdot ,S_{k}$, the probability $P_{S_{1},S_{n}}$ of each possible next position $S_{n}$ can be calculated by using the weighted Markov model:
  (16) $$P_{S_{1},S_{n}}=\sum_{j=1}^{k}\left(\omega_{j}P_{S_{j},S_{n}}^{\left(j\right)}\right),$$
  where $P_{S_{j},S_{n}}^{\left(j\right)}$ represents the transition probability of the user from location $S_{j}$ through *j* steps to $S_{n}$, and $\omega_{1},\omega_{2},\cdot \cdot \cdot ,\omega_{k}$ are the weighting coefficients of different steps.
- Predicting the user’s next location: The probabilities of all possible locations are compared. $S_{n}$, which corresponds to the maximum probability, is the predicted location of the user at the next moment.

### 5.3. Parameter Optimization of the Weighted Markov Prediction Model

**Figure 3:**
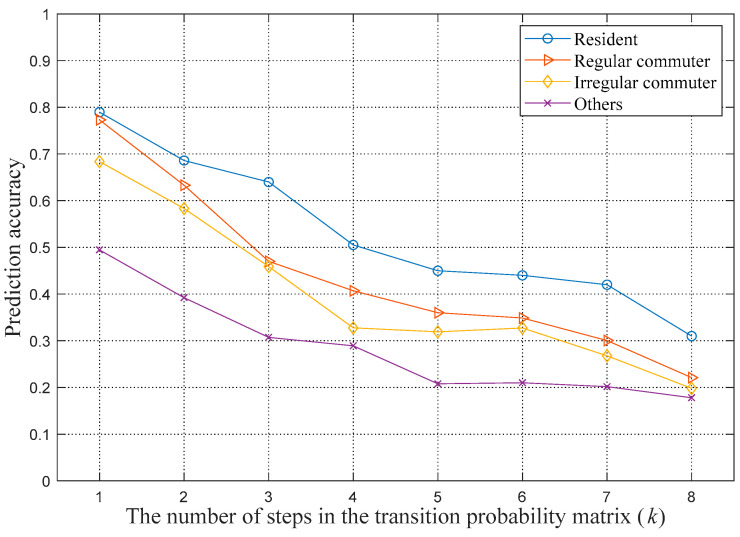
Prediction accuracy of Markov models based on different steps *k*.

To evaluate the proposed weighted Markov prediction model based on different user classifications, corresponding tests and analyses are performed in this paper. First, the optimal number of steps *k* and the weighting coefficient of each step need to be determined. The value of *k* is determined according to the step threshold $L_{th}$ and the probability threshold, $P_{th}$, and the weighting coefficient of each step is determined according to the single step transition probability. The step threshold $L_{th}$ is set to 8 in our experiment. To determine an optimal probability threshold, the prediction accuracy of each step to the next location is calculated based on the 1- to 8-step probability transition matrices. The accuracy can explain the influence degree of different locations on the next location. The results are shown in Figure 3.

From Figure 3, we observe that the accuracy of the prediction of the next location decreases as the step size increases. That is, the location closer to the prediction time has a greater degree of influence on the prediction. Based on the actual situation and the evaluation of the computational complexity, the probability threshold $P_{th}$ is set to 30%.

**Figure 4:**
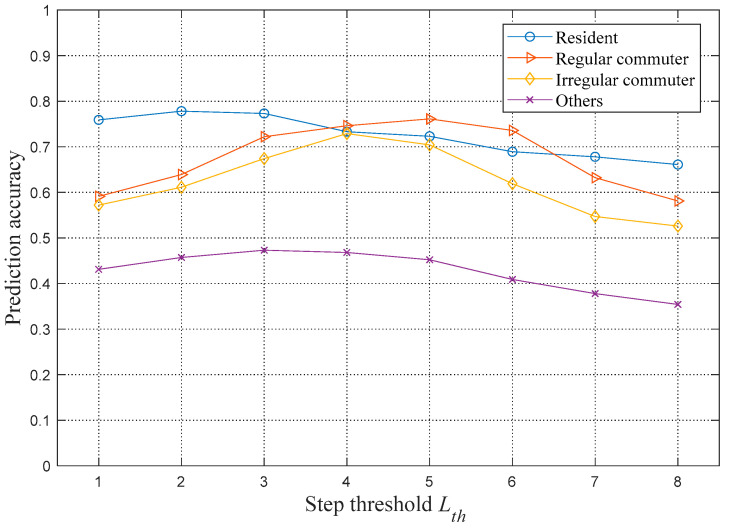
Relationship between the step threshold and the prediction accuracy.

In addition, to determine the optimal number of steps, this paper selects 50 users in each classification and calculates the prediction accuracy under different numbers of steps. The results are shown in Figure 4. It is observed that with the increase in the number of steps, the prediction accuracies of various types of users will first increase and then decrease. This observation shows that the first few locations before the prediction moment will have more important effects on the prediction. Moreover, for different types of users, the peak value of prediction accuracy appears at different steps. The variation in the prediction accuracy of residents is minimal, and the peak value of accuracy appears at a step threshold of 2. The regular and irregular commuters have similar trends in prediction accuracy, with peaks at step thresholds of 5 and 4, respectively. The peak value of the prediction accuracy of others appears at a step threshold of 3.

Therefore, according to the prediction accuracy of different steps, different weighting coefficients are taken for different types of users. Table 6 shows the optimal step sizes of weighted Markov models for various user types. It is observed that the maximum step of regular commuters is 5, which means that the corresponding Markov order is also 5. Although this is a relatively high order, the overall complexity of the algorithm does not increase significantly because this type of users only account for about one-third of all users, while the orders of other types of users are relatively low. After the optimal step number is determined, the weighting coefficients of different steps can be calculated according to Equation (15), and the results are shown in Table 7.

### 5.4. Analysis of Prediction Results

Based on the user classification results, 1000 users are randomly selected from each type of user. The first 14 days of the trajectory data of the users are selected as training samples, and the trajectory data of the next 7 days are selected as prediction samples. Location prediction is performed for each user, and the prediction results are compared with the actual record to determine the prediction accuracy.

**Figure 5:**
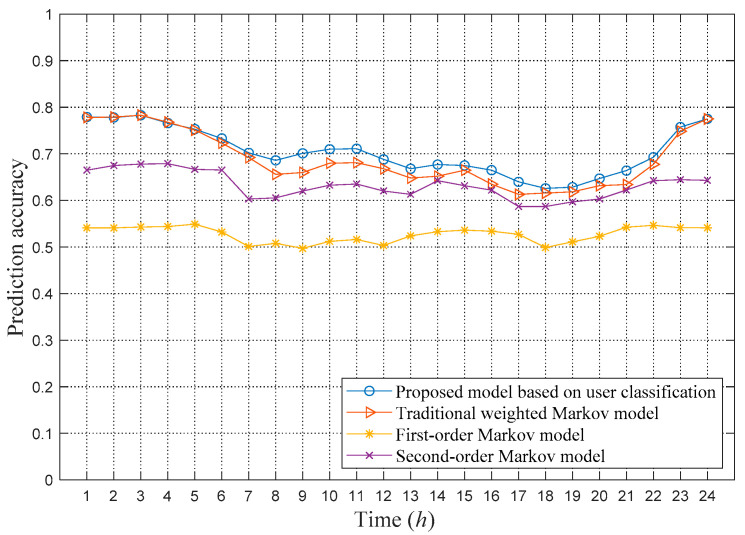
Comparison of prediction accuracy of different Markov models.

Based on the optimal step threshold and weighting coefficients determined in the previous section, the weighted Markov model is used to predict the next location of four types of users, and the prediction accuracy is calculated in units of one hour. The prediction accuracy of the weighted Markov model based on different user types proposed in this paper is compared with the prediction accuracy of traditional weighted Markov models and first- and second-order Markov prediction models. Among them, the traditional weighted Markov model does not perform classification processing for all users. The highest order of the traditional Markov model is set to 4, and the weighting coefficients are calculated in a decreasing manner as given in Equation (13). In the second-order Markov model, the effect of the two locations before the current location on the transition probability is considered. The test results are shown in Figure 5.

Figure 5 shows that the proposed model based on user classification has the highest prediction accuracy, reaching an average of 70.4%. The first-order Markov model has the lowest prediction accuracy, with an average of only 52.7%, which indicates that it is insufficient to consider only the current position when predicting the next location. The accuracies of the traditional weighted Markov and second-order Markov models are greater than the prediction accuracy of the first-order Markov model, which indicates that it is beneficial to the prediction to fully consider the past locations. However, the prediction accuracy of the traditional weighted model is lower than the prediction accuracy of the proposed weighted Markov model, and it uses a higher order for all users to predict, which requires more computing resources.

**Figure 6:**
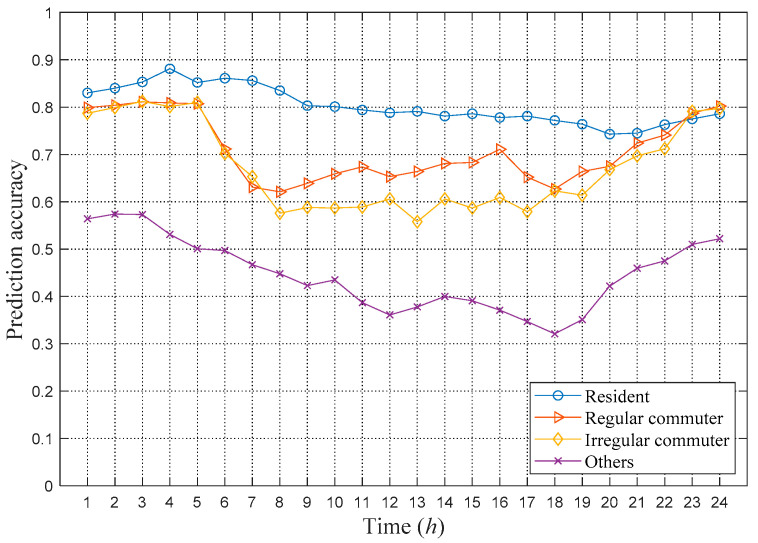
Prediction results of the weighted Markov model based on different user classifications.

In addition, Figure 6 shows the distribution of the weighted Markov prediction accuracy for each type of user against different times of the day. Figure 6 shows that the prediction accuracy has a negative correlation with user mobility. Residents have the lowest mobility, so their prediction accuracy is the highest. For regular commuters, their prediction accuracy during the morning and evening peak periods is relatively low. For irregular commuters, the accuracy of their working hours during the day is relatively low. The prediction accuracy of the category of others is very low overall. Even during the night, the category of others is still much lower than other types of users. These are due to that higher mobility resulting in more states in the Markov transition matrix, which scatter the influence of historical trajectories on the prediction of the next location. For example, for a user category that includes couriers or truck drivers, the nature of their work leads to irregular mobility, which makes the next location almost impossible to predict. Therefore, the more the user moves, the higher the uncertainty in the user mobility, which leads to a lower prediction accuracy.

### 5.5. Discussion and Future Work

**Figure 7:**
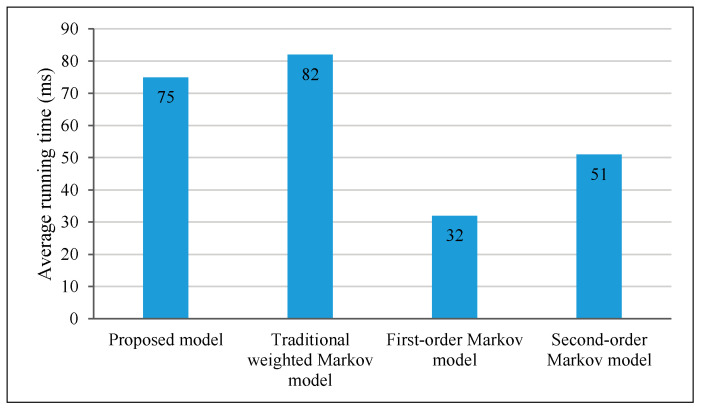
Prediction results of the weighted Markov model based on different user classifications.

The complexity of Markov models is an important parameter in measuring the performance, which usually affects the practical application of the algorithm. Therefore, we assessed the average running time of each model, as shown in Figure 7. It should be noted that these data are calculated by using MATLAB R2016a on the personal computer equipped with Intel i5-7200u CPU and 8G memory. Figure 7 shows that the average running time of the proposed model is slightly lower than that of the traditional weighted Markov model because it adopts lower orders in some specific user types. The average running time of the first- and second-order Markov models is much lower because of the lower order.

Although the weighted Markov model proposed in this paper improves the trajectory prediction accuracy of users to a certain extent, the magnitude of the improvement is not obvious. Especially in the period of high mobility or for users with irregular mobility, the prediction accuracy has a significant decline. To further improve the prediction accuracy, in the future, we will mainly improve the research from the following aspects. First, different classification criteria can be tested, or users can be clustered using an unsupervised model. Second, to evaluate the classification accuracy of mobile users by different machine learning algorithms, more machine learning algorithms can be tested and the parameters of the algorithms can be optimized to obtain more accurate classification. Last, the Markov chain-based prediction methods can be compared with the dynamic time warping (DTW) algorithms and neural network-based prediction methods, so as to reveal the difference between different types of prediction algorithms.

## 6. Conclusions

Smartphones have become an essential part of daily life for billions of people. Together with the continuous advancement of mobile communication technology, mobile user trajectory information mining has attracted increasing attention in the field of data mining. At present, location prediction technology is still immature, with problems such as low accuracy and a large amount of processing requirements, and it is challenging to design useful applications that can help service providers gain value. To address the problems of traditional Markov models in location prediction, a weighted Markov location prediction model based on different user classifications is proposed in this paper. This model uses different weighted Markov models with different parameters for different types of users, and it achieves higher prediction accuracy than traditional models.

The data collected from mobile networks regarding the cell sites accessed by mobile users are used to predict the location of these users. The cell sites are densely distributed in cities but sparsely distributed in less densely populated scenes such as suburbs and rural areas. Therefore, the proposed location prediction method is more suitable for urban users, and the prediction accuracy for users in remote suburbs and counties is expected to be lower than that of urban users. However, with the subsequent deployment and commercialization of 5G networks, the deployment of cellular base stations in various places will become denser. This outcome will enable our proposed model to be widely used in the 5G era. In addition, the classification method proposed in this paper based on mobile characteristics could be extended to include other features in future work, such as service preferences, browsing web content, and traffic characteristics. This future research can then be used to further explore the similarities and differences between different users. As a result, we show that a more accurate user classification model can be constructed, and more accurate predictions of user mobility and network resource requirements can be achieved.

## Figures and Tables

**Table 1:** User types and characteristics.

User Type	Description
Residents	People who do not move or move very little. They often visit only a few fixed locations. For example, retirees, housewives, etc.
Regular commuters	They have regular movement patterns of office workers on weekdays. They have strong mobility in the morning and evening hours, weak mobility during the day and night, and have fixed working and living places.
Irregular commuters	They have strong mobility during the day and have no obvious regularity, but they have weak mobility at night.
Others	People who do not meet the three characteristics above. For example, people with high mobility at night.

**Table 2:** Parameter name and corresponding description.

Parameter Name	Description
cellNum	The number of unique cell sites visited in one day.
cellNum_amPeak	The average number of unique cell sites is included in the trajectory during the morning peak (7:00–10:00) every day.
cellNum_pmPeak	The average number of unique cell sites is included in the trajectory during the evening peak (17:00–21:00) every day.
cellNum_dayPeakoff	The average number of unique cell sites is included in the trajectory during the working time (10:00–17:00) every day.
cellNum_nightPeakoff	The average number of unique cell sites is included in the trajectory during the evening time (21:00–23:00) every day.
cellNum_sleep	The average number of unique cell sites is included in the trajectory during the night sleeping time (23:00–7:00) every day.
residLen_dayPeakoff	The average length of resident time during the working time (10:00–17:00) every day. This is measured by the dwell time of a fixed cell.
residLen_night	The average length of resident time during the night (0:00–7:00) every day. This is measured by the dwell time of a fixed cell.

**Table 3:** Classification criteria of each user type.

User Type	Classification Criteria
Residents	cellNum ≤ 5
Regular commuters	cellNum > 5 & cellNum_amPeak ≥ 2 & cellNum_pmPeak ≥ 2 & cellNum_dayPeakoff < 3 & cellNum_sleep ≤ 3 & residLen_dayPeakoff ≥ 300 & residLen_night ≥ 360
Irregular commuters	cellNum > 5 & cellNum_dayPeakoff ≥ 3 & cellNum_nightPeakoff ≥ 2 cellNum_sleep > 3 & residLen_night < 360
Others	Others

**Table 4:** Comparison of evaluation indicators based on different classification models.

Machine Learning Algorithms	Evaluation Indicators	Residents	Regular Commuters	Irregular Commuters	Others
Naive Bayes	Precision	88.24%	84.00%	79.17%	78.57%
	Recall	93.75%	87.50%	79.17%	68.75%
	F1-score	90.91%	85.71%	79.17%	73.33%
	Accuracy	82.50%			
Decision Tree	Precision	100%	92.00%	87.50%	100%
	Recall	100%	95.83%	87.50%	85.71%
	F1-score	100%	93.88%	87.50%	92.31%
	Accuracy	92.50%			
KNN	Precision	100%	92.00%	91.67%	100%
	Recall	100%	95.83%	91.67%	93.75%
	F1-score	100%	93.88%	91.67%	96.77%
	Accuracy	95.00%			

**Table 5:** User classification results.

User Type	Number of Users	Proportion
Residents	1092	22.21%
Regular commuters	1671	33.98%
Irregular commuters	1075	21.86%
Others	1079	21.95%

**Table 6:** Maximum steps for different types of users.

User Type	Maximum Step (*k*)
Residents	2
Regular commuters	5
Irregular commuters	4
Others	3

**Table 7:** Weighting coefficients of different steps for different types of users.

User Type	Weighting Coefficient				
	One-Step	2-Step	3-Step	4-Step	5-Step
Residents	0.535	0.465	-	-	-
Regular commuters	0.292	0.240	0.178	0.154	0.136
Irregular commuters	0.333	0.284	0.178	0.154	-
Others	0.414	0.328	0.257	-	-

## Data Availability

All data generated or analyzed during this study are included in this published article.
